# Psychosocial risks and work ability among health workers throughout the COVID-19 pandemic: a longitudinal follow-up study, Brazil, 2020-2023

**DOI:** 10.1590/S2237-96222026v35e20240798.en

**Published:** 2026-03-09

**Authors:** Carolina Luciane Nogueira Martinez, Cristiane Shinohara Moriguchi, Marcela Alves Andrade, Tatiana de Oliveira Sato

**Affiliations:** 1 Universidade Federal de São Carlos, Centro de Ciências Biológicas e da Saúde, Programa de Pós-Graduação em Fisioterapia, São Carlos, SP, Brazil; 2 Universidade Federal de São Carlos, Centro de Ciências Biológicas e da Saúde, Departamento de Fisioterapia, São Carlos, SP, Brazil

**Keywords:** SARS-CoV-2, Occupational Risks, Psychosocial Impact, Work Capacity Evaluation, Longitudinal Studies, SARS-CoV-2, Riesgos Laborales, Impacto psicosocial, Evaluación de Capacidad de Trabajo, Estudios longitudinales

## Abstract

**Objective:**

To compare health workers and non-health-related workers in terms of psychosocial risks and work capacity over a 36-month follow-up period.

**Methods:**

This was a prospective longitudinal study. Participants completed a sociodemographic questionnaire, the Copenhagen Psychosocial Questionnaire II, and the work capacity index. Data were analyzed descriptively using absolute and relative frequencies and 95% confidence intervals (95%CI). Chi-square and Cochran’s tests were used for inter- and intra-group comparisons.

**Results:**

A total of 1,211 workers participated in 2020, including 219 health workers and 992 non-health-related workers . In that year, three out of four health workers experienced high emotional demands (75.8%; 95%CI 69.7; 81.0%), poor self-rated health (14.6%; 95%CI 10.5; 19.9%), burnout (83.1%; 95%CI 77.6; 87.5%), unwanted sexual attention (11.0%; 95%CI 7.5; 15.8%), threats of violence (19.6%; 95%CI 14.9; 25.4%), and physical violence (2.3%; 95%CI 1.0; 5.2%). In 2023, emotional demands (71.4%; 95%CI 60.5; 80.3%) and threats of violence (18.2%; 95%CI 11.1; 28.2%) remained elevated. Work capacity remained stable in both groups. The frequency of classification was good in 2020 (44.3 and 49.9), 2021 (52.5 and 46.2), 2022 (45.7 and 50.4), and 2023 (46.6 and 43.3) among health workers and non-health-related workers , respectively.

**Conclusion:**

Healthcare professionals were exposed to psychosocial risks during the pandemic, mainly emotional demands and threats of violence, which indicates a need for intervention in the workplace.

## Resumen

**Objetivo:**

Comparar a los profesionales sanitarios y a los profesionales no relacionados con la asistencia sanitaria en cuanto a los riesgos psicosociales y la capacidad para el trabajo durante 36 meses de seguimiento.

**Métodos:**

Se trata de un estudio longitudinal prospectivo. Los participantes respondieron a un cuestionario sociodemográfico, al Cuestionario Psicosocial de Copenhague II y al índice de capacidad para el trabajo. Los datos se analizaron de forma descriptiva mediante frecuencias absolutas y relativas y un intervalo de confianza del 95 % (IC95 %). Se utilizaron pruebas de chi cuadrado y Cochran para la comparación entre grupos y dentro de los mismos.

**Resultados:**

Participaron 1211 trabajadores (219 profesionales sanitarios y 992 profesionales no relacionados con la asistencia sanitaria) en 2020. Ese año, tres de cada cuatro profesionales sanitarios se enfrentaron a altas exigencias emocionales (75,8 %; IC95 % 69,7; 81,0 %), mala percepción de la salud (14,6 %; IC95 % 10,5; 19,9 %), agotamiento (83,1 %; IC95 % 77,6; 87,5 %), atención sexual no deseada (11,0 %; IC95 % 7,5; 15,8 %), amenazas de violencia (19,6 %; IC95 % 14,9; 25,4 %) y violencia física (2,3 %; IC95 % 1,0; 5,2 %). En 2023, las demandas emocionales (71,4 %; IC95 % 60,5; 80,3 %) y las amenazas de violencia (18,2 %; IC95 % 11,1; 28,2 %) siguieron siendo mayores. La capacidad para el trabajo se mantuvo estable en ambos grupos. La frecuencia de la clasificación fue buena en 2020 (44,3 y 49,9), 2021 (52,5 y 46,2), 2022 (45,7 y 50,4) y 2023 (46,6 y 43,3) en los profesionales sanitarios y en los profesionales no relacionados con la asistencia sanitaria.

**Conclusiones:**

: Los profesionales sanitarios estuvieron expuestos a riesgos psicosociales durante la pandemia, principalmente a demandas emocionales y amenazas de violencia, lo que indica la necesidad de intervenir en el entorno laboral.

**Ethical aspects**

This research respected ethical principles, having obtained the following approval data:

Research ethics committee: Universidade Federal de São Carlos

Opinion number: 4166321

Approval date: 21/7/2020

Certificate of submission for ethical appraisal: 31885020.9.0000.5504

Informed consent record: Obtained from all participants prior to data collection.

## Introduction

Psychosocial risks related to work result from the interaction between workplace factors and human factors, and may influence health, performance, and work ability ^1^. The COVID-19 pandemic changed working conditions, making it necessary to monitor its effects on psychosocial risks and work ability ^2^ ^,^ ^3^.

Health professionals faced a variety of challenges during this period. Changes in the work environment, such as increased workload, conflicting demands, lack of social support, long shifts, and rotating schedules, contributed to a rise in risk factors among these professionals ^4^ ^,^ ^5^. The pandemic had direct effects on health workers, including increased levels of anxiety, depression, insomnia, post-traumatic stress, and burnout ^5^. 

Exhausting work routines were also present in other sectors. Those performing essential roles, for example, supermarket cashiers and delivery workers, faced emotional exhaustion, increased risk of infection, and deteriorating working conditions during the pandemic period in Brazil ^6^ ^,^ ^7^. In general, the pandemic affected the mental health of the adult population, with a 52.6% prevalence of anxiety symptoms found in a survey of 45,000 Brazilians in 2020 ^8^. However, health professionals showed higher prevalence of depression, acute stress, and burnout compared to the general population ^4^ ^,^ ^9^. It is suggested that the psychosocial risk factors to which these workers were exposed during the pandemic were also greater than those experienced by the general population. 

A six-month longitudinal follow-up study of Brazilian health workers showed a decline in mental health during the first year of the pandemic ^10^. In Portugal, the mental health of health workers was monitored over a two-year period, and findings showed the persistence of burnout symptoms and a decrease in anxiety and post-traumatic stress symptoms from 2020 to 2021 ^11^. 

Work ability was also affected by social restrictions and changes in work arrangements during the pandemic ^3^. Work ability results from the balance between workers’ physical and mental resources and job demands, and can be assessed using the Work Ability Index ^12^.

This study aimed to compare health workers and non-health-related workers in terms of psychosocial risks and work ability over a 36-month follow-up period after the onset of the COVID-19 pandemic in Brazil. Although illness among health professionals has been widely studied, this study has made progress in identifying psychosocial risk factors and in longitudinal follow-up.

## Methods

Study Design

This was a longitudinal cohort study with a 36-month prospective follow-up. Data were collected every 12 months in 2020 (baseline), 2021, 2022, and 2023. 

Setting

The study was conducted online and publicized through local press announcements, social media, and email invitations. With national coverage, it was initially promoted by the university’s Office of Social Communication and later disseminated through other media outlets, including websites, radio stations, and television channels. Both the social media platforms and email addresses used to send the invitations were institutional. Email addresses were obtained from public websites and sent through the researchers’ contact networks. 

Participants

The study was part of the project “Implications of the COVID-19 pandemic on psychosocial aspects and work ability among Brazilian workers,” which has been assessing Brazilian workers annually since 2020 in terms of psychosocial factors and work ability ^2^.

Variables

Participants completed a sociodemographic questionnaire, the work ability index ^12^, and the short version of the Copenhagen Psychosocial Questionnaire II (COPSOQ II-Br) ^13^.

Data sources

Workers who agreed to participate in the study, were aged 18 years or older, resided in Brazil, and worked in any professional sector were included. Students, interns, retirees, and individuals with duplicate records were not included. Health professionals comprised nurses, physical therapists, pharmacists, speech therapists, physicians, veterinarians, nutritionists, physical educators, psychologists, nursing technicians, health sector administrative staff, and faculty in health-related college/university programs.

In the initial assessment, workers filled in a sociodemographic and occupational questionnaire, a short version of COPSOQ II-Br and the work ability index. In the following years, participants were contacted by email and invited to respond the COPSOQ II-Br and the work ability index. Assessments were conducted between June and September each year (2020-2023), totaling four rounds of data collection.

The sociodemographic and occupational questionnaire contained questions related to sex, age, race/skin color, city, state, marital status, number of children, weight, height, smoking status, medication use, education, occupation, length of time in occupation, type of current employment, professional sector, family income, employment status, job loss during the pandemic, and questions about the COVID-19 pandemic (e.g., whether they contracted COVID-19, received vaccines, used personal protective measures, etc.). 

The COPSOQ II-Br was used to identify psychosocial risks in the workplace ^13^. This instrument adopts a multifaceted approach, comprising 23 dimensions and 40 items ^14^. It identifies the following risk factors: quantitative demands, work pace, emotional demands, opportunities for development, meaning of work, commitment, recognition, social support, job satisfaction, work-family conflict, burnout, stress, and offensive behaviors (unwanted sexual attention, threats of violence, physical violence, and bullying) ^13^. The COPSOQ II-Br was translated and culturally adapted for use in the Brazilian population ^13^.

Each item was rated on a five-point response scale. Item 1B was the only one with reverse scoring. Scores for each dimension were obtained by summing the respective item responses, with the exception of the offensive behaviors dimension. Higher total scores reflected higher psychosocial risk levels.

The work ability index, translated into Brazilian Portuguese, evaluates workers’ perception of their work ability considering health and job demands ^12^. It does not require an interviewer and consists of seven dimensions: current work ability compared to the best ever, work ability in relation to job demands, number of physician-diagnosed current diseases, estimated work impairment due to diseases, sick leave in the past 12 months, self-prognosis of work ability in two years, and mental resources. 

Scores on the work ability index ranged from 7 to 49, with the following classifications: low (7-27), moderate (28-36), good (37-43), and excellent (44-49) ^12^.

Bias

The study’s dissemination strategy and mode of participant contact may have introduced selection bias, as individuals with limited internet access or lower digital literacy were underrepresented in the sample.

Study Size

All individuals who met the inclusion criteria and responded to the survey were considered for analysis; no group balancing was performed.

Statistical methods

The sociodemographic profile, psychosocial risks, and work ability were described using absolute and relative frequencies and 95% confidence intervals (95%CI) for categorical variables. Mean, standard deviation, minimum, and maximum were calculated for continuous variables. Comparisons between the health workers group and the non-health-related workers group were performed using the chi-square test. The comparison between assessment periods, conducted separately for each group, was performed using Cochran’s test. The significance level was 5%.

## Results

**Figure 1.**
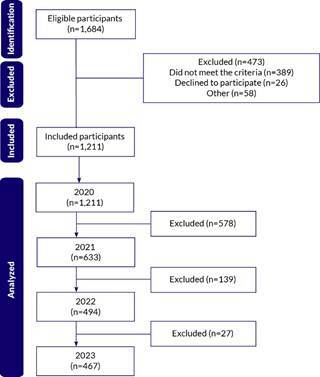
Number of participants during the study follow-up. São Carlos, 2020-2023 (n=1,211)

A total of 1,211 workers were included in 2020, comprising 219 health workers and 992 non-health-related workers (Figure 1). The mean age of workers in 2020 was 37.7 years (standard deviation 10.5), with no significant difference (p-value 0.125) between health workers (mean 36.7; SD 9.5) and non-health-related workers (mean 37.9; SD 10.8). The group of health workers had a higher proportion of women (75.8%), university education (88.1%), lower proportion of people working from home (42.9%), higher proportion of coronavirus infection (10.5%), and higher proportion of workers with income between 3 and 12 minimum wages (67.6%) (Table 1). Other variables were similar between the groups. 

**Table 1:** Relative frequency (%) and 95% confidence interval (95%CI) of sociodemographic and occupational characteristics at baseline according to groups of health workers (n=219) and non-health-related workers (n=992). São Carlos, 2020 (n=1,211)

Characteristic	Total	Health workers (n=219)% (95%CI)	Non-health-related workers (n=992)% (95%CI)	p-value
	(N=1,211)% (95%CI)			
Sex				
Female	51.9 (49.1; 54.7)	75.8 (69.7; 81.0)	46.7 (43.6; 49.8)	<0.001
Male	48.1 (45.3; 50.9)	24.2 (19.0; 30.3)	53.3 (50.2; 56.4)	
**Marital status**				0.664
Single/widowed/divorced	45.3 (42.5; 48.1)	46.6 (40.1; 53.2)	45.0 (41.9; 48.1)	
Married/Common-law marriage	54.7 (51.9; 57.5)	53.4 (46.8; 59.9)	55.0 (51.9; 58.1)	
**Education level**				0.030
Undergraduate	83.2 (80.9; 85.1)	88.1 (83.2; 91.8)	82.1 (79.5; 84.3)	
Not undergraduate	16.8 (14.8; 19.1)	11.9 (8.2; 16.8)	17.9 (15.7; 20.4)	
Smoker	5.8 (4.6; 7.2)	5.5 (3.2; 9.3)	5.8 (4.5; 7.5)	0.582
Employment contract				0.247
Public servant	41.0 (38.2; 43.7)	39.3 (33.0; 45.9)	41.4 (38.3; 44.4)	
Private sector employee	44.0 (41.2; 46.8)	41.1 (34.8; 47.7)	44.7 (41.6; 47.8)	
Informal	0.2 (0.0; 0.6)	0.5 (0.1; 2.5)	0.5 (0.0; 0.6)	
Independent contractor	0.8 (0.4; 1.5)	0.9 (0.2; 3.3)	0.8 (0.4; 1.6)	
Entrepreneur	0.7 (0.4; 1.4)	0.5 (0.1; 2.5)	0.8 (0.4; 1.6)	
**Professional sector**				<0.001
Health	18.1 (16.0; 20.3)	100 (98.3; 100.0)	-	
Education	37.2 (34.5; 39.9)	-	45.4 (42.3; 48.5)	
Essential business	3.3 (2.4; 4.5)	-	4.0 (3.0; 5.4)	
Non-essential business	5.9 (4.7; 7.3)	-	7.2 (5.7; 8.9)	
Civil Construction	2.1 (1.5; 3.1)	-	2.6 (1.8; 3.8)	
Public safety	1.3 (0.8; 2.4)	-	1.6 (1.0; 2.3)	
Public transportation	0.3 (0.1; 0.8)	-	0.4 (0.2; 1.0)	
Agriculture	1.2 (0.7; 2.0)	-	1.5 (0.9; 2.5)	
Private transportation	0.3 (0.1; 0.8)	-	0.4 (0.2; 1.0)	
Delivery service	0.2 (0.0; 0.6)	-	0.2 (0.1; 0.7)	
Cleaning	0.1 (0.0; 0.5)	-	0.1 (0.0; 0.6)	
Logistics services	4.0 (3.0; 5.2)	-	4.8 (3.7; 6.4)	
Industry	6.8 (5.5; 8.3)	-	8.3 (6.7; 10.1)	
Public sector	5.3 (4.2; 6.7)	-	6.5 (5.1; 8.1)	
Non-governmental organizations	0.3 (0.1; 0.8)	-	0.4 (0.2; 1.0)	
Banking	1.5 (0.9; 2.3)	-	1.8 (1.1; 2.8)	
IT	7.8 (5.1; 7.9)	-	7.8 (6.3; 9.6)	
Law practice	3.6 (2.6; 4.7)	-	4.3 (3.2; 5.8)	
Communication	1.8 (1.2; 2.7)	-	2.2 (1.5; 3.3)	
Tourism and hospitality	0.4 (0.2; 1.0)	-	0.4 (0.2; 1.1)	
**Working from home**	74.1 (71.5; 76.5)	42.9 (36.5; 49.5)	80.9 (78.4; 83.3)	<0.001
**Coronavirus infection**	5.4 (4.2; 6.8)	10.5 (7.1; 15.3)	4.2 (3.1; 5.7)	<0.001
Race/skin color				0.556
White	49.3 (46.5; 52.1)	42.9 (36.5; 49.5)	50.7 (47.6; 53.8)	
Non-white	5.7 (2.1; 4.1)	3.2 (1.6; 6.4)	5.5 (2.0; 4.1)	
**Use of medication**	40.4 (37.5; 43.0)	41.1 (34.8; 47.7)	40.2 (37.0; 43.1)	0.769
**Monthly family income (minimum wage)**				0.017
Not declared	5.0 (3.9; 6.3)	2.3 (1.0; 5.2)	5.5 (4.3; 7.1)	
Up to 1	1.7 (1.1; 2.5)	1.4 (0.5; 3.9)	1.7 (1.1; 2.7)	
01/mar	18.0 (15.9; 20.3)	15.5 (11.3; 20.9)	18.5 (16.2; 21.1)	
03/jun	26.6 (24.2; 29.1)	31.5 (25.7; 37.9)	25.5 (22.9; 28.3)	
06/set	16.0 (14.1; 18.2)	21.0 (16.1; 26.9)	14.9 (12.8; 17.3)	
09/dez	14.4 (12.5; 16.5)	15.1 (10.9; 20.4)	14.2 (12.2; 16.5)	
>12	18.4 (16.3; 20.7)	13.2 (9.4; 18.4)	14.2 (17.2; 22.1)	
Body mass index				0.614
**Underweight**	1.5 (0.9; 2.3)	1.8 (0.7; 4.6)	1.4 (0.8; 2.3)	
Normal	39.2 (36.5; 42.0)	42.0 (35.7; 48.6)	38.6 (35.6; 41.7)	
Overweight	35.7 (33.0; 38.4)	35.6 (29.6; 42.1)	35.7 (32.8; 38.7)	
**Obese**	23.6 (21.3; 26.1)	20.5 (15.7; 26.4)	24.3 (21.7; 27.1)	

Significant differences between the groups were identified regarding psychosocial risks (Table 2). Throughout the follow-up period, health professionals consistently exhibited a higher risk for emotional demands (p-value<0.050). Differences between the groups were observed in the dimensions of general health perception, burnout, unwanted sexual attention, threats of violence, and physical violence; in 2020 and 2021, health professionals were at greater risk.

Among non-health-related workers, higher risk levels were found for the following dimensions: influence at work (2020 and 2023), opportunities for development (2020, 2021, and 2022), meaning of work (2020, 2021, 2022, and 2023), commitment to work (2020 and 2021), and clarity of roles and responsibilities (2022).

Significant differences were observed in the “influence at work” dimension over time, with increasing risk among health professionals from 2020 to 2022, followed by a decrease in 2023. Differences between 2020-2022 (p-value 0.040), 2021-2023 (p-value 0.040), and 2022-2023 (p-value 0.003) were statistically significant. During the pandemic, the predictability risk factor also increased in 2021 (p-value 0.010), 2022 (p-value 0.022), and 2023 (p-value 0.022) compared to 2020. 

Among non-health-related workers, a reduction in the influence at work risk was found when comparing 2020 to 2022 (p-value 0.005) and 2020 to 2023 (p-value 0.018), and a reduction in trust in leadership was observed between 2021 and 2023 (p-value 0.004). On the other hand, these professionals experienced an increase in the meaning of work risk from 2020 to 2021 (p-value 0.001), 2022 (p-value 0.020), and 2023 (p-value 0.001). 

**Table 2:** Relative frequency (%) and 95% confidence interval (95%CI) of psychosocial aspects related to work among health workers (health) and non-health-related workers (non-health). São Carlos, 2020-2023 (n=1,211)

Dimensions	2020		2021		2022		2023	
	Health (n=219)% (95%CI)	Non-health (n=992)% (95%CI)	Health (n=101)% (95%CI)	Non-health (n=532)% (95%CI)	Health (n=81)% (95%CI)	Non-health (n=413)% (95%CI)	Health (n=77)% (95%CI)	Non-health (n=390)% (95%CI)
Quantitative demands	9.6	9.7	10.9	12.4	18.5	13.3	10.4	14.9
	(6.4; 14.2)	(8.0; 11.7)	(6.2; 8.5)	(9.9; 15.5)	(11.6; 28.3)	(10.4; 16.9)	(5.4; 19.2)	(11.7; 18.7)
Work pace	51.6	48.6	49.5	46.8	49.4	42.6	50.6	45.1
	(45.0; 58.1)	(45.5; 51.7)	(39.9; 59.1)	(42.6; 51.0)	(38.8; 60.0)	(37.9; 47.4)	(39.7; 61.5)	(40.3; 50.0)
Emotional demands	75.8^a^	48.8	75.2^a^	43.2	67.9^a^	48.2	71.4 ^a^	49.7
	(69.7; 81.0)	(45.7; 51.9)	(66.0; 82.6)	(39.1; 47.5)	(57.1; 77.1)	(43.4; 53.0)	(60.5; 80.3)	(44.8; 54.7)
Influence at work	25.6	36.1 ^a^	27.7	33.1	29.6	28.6	16.9	29.7 ^a^
	(20.2; 31.7)	(33.2; 39.1)	(19.9; 37.1)	(29.2; 37.2)	(20.8; 40.3)	(24.4; 33.1)	(10.1; 26.8)	(25.4; 34.5)
Opportunities for development	2.3	8.6 ^a^	2.0	7.3 ^a^	-	7.5 ^a^	2.6	8.5
	(1.0; 5.2)	(7.0; 10.5)	(0.5; 6.9)	(5.4; 9.9)		(5.3; 10.5)	(0.7; 9.0)	(6.1; 11.6)
Meaning of work	6.8	12.2 ^a^	6.9	16.7 ^a^	4.9	14.8 ^a^	6.5	14.9 ^a^
	(4.2; 11.0)	(10.3; 14.4)	(3.4; 13.6)	(13.8; 20.1)	(1.9; 12.0)	(11.7; 18.5)	(2.8; 14.3)	(11.7; 18.7)
Commitment to work	6.8	12.2 ^a^	5.9	13.9 ^a^	7.4	14.5	13.0	13.8
	(4.2; 11.0)	(10.3; 14.4)	(2.7; 12.4)	(11.2; 17.1)	(3.4; 15.2)	(11.5; 18.2)	(7.2; 22.3)	(10.8; 17.6)
Predictability	24.7	26.1	27.7	26.5	22.2	24.9	27.3	24.1
	(19.4; 30.8)	(23.5; 28.9)	(19.9; 37.1)	(22.9; 30.4)	(14.5; 32.4)	(21.0; 29.3)	(18.6; 38.1)	(20.1; 28.6)
Recognition of work	19.2	17.9	15.8	16.9	16.0	18.6	19.5	16.4
	(14.5; 24.9)	(15.7; 20.4)	(10.0; 24.2)	(14.0; 20.3)	(9.6; 25.5)	(15.1; 22.7)	(12.1; 29.7)	(13.1; 20.4)
Clarity of roles and responsibilities	11.4	10.6	10.9	12.6	4.9	14.3 ^a^	15.6	13.1
	(7.8; 16.3)	(8.8; 12.6)	(6.2; 18.5)	(10.0; 15.7)	(1.9; 12.0)	(11.2; 18.0)	(9.1; 25.3)	(10.1; 16.8)
Leadership quality	28.8	26.2	25.7	23.1	21.0	25.4	32.5	26.2
	(23.2; 35.1)	(23.6; 29.0)	(18.2; 35.0)	(19.7; 26.9)	(13.5; 31.1)	(21.5; 29.8)	(23.1; 43.5)	(22.0; 30.7)
Social support from supervisors	19.6	21.1	17.8	18.4	17.3	20.6	23.4	21.8
	(14.9; 25.4)	(18.6; 23.7)	(11.6; 26.4)	(15.4; 21.9)	(10.6; 26.9)	(17.0; 24.7)	(15.3; 34.0)	(18.0; 26.1)
Work satisfaction	17.4	17.9	11.9	18.8	16.0	17.9	24.7	18.7
	(12.9; 22.9)	(15.7; 20.4)	(6.9; 19.6)	(15.7; 22.3)	(9.6; 25.5)	(14.5; 21.9)	(16.4; 35.3)	(15.1; 22.9)
Work-family conflict	47.0	46.1	45.5	47.0	40.7	45.0	45.5	49.7
	(40.5; 53.6)	(43.0; 49.2)	(36.2; 55.2)	(42.8; 51.2)	(30.7; 51.6)	(40.3; 49.9)	(34.8; 56.5)	(44.8; 54.7)
Trust in leadership	9.6	9.5	10.9	7.0	6.2	10.4	15.6	11.5
	(6.4; 14.2)	(7.8; 11.5)	(6.2; 18.5)	(5.1; 9.4)	(2.7; 13.6)	(7.8; 13.7)	(9.1; 25.3)	(8.7; 15.1)
Justice and respect	23.3	19.7	18.8	19.2	19.8	20.3	28.6	21.8
	(18.2; 29.3)	(17.3; 22.2)	(12.4; 27.5)	(16.0; 22.7)	(12.5; 29.7)	(16.7; 24.5)	(19.7; 39.5)	(18.0; 26.1)
Self-rated health	14.6 ^a^	9.8	10.9	13.0	12.3	9.2	13.0	11.0
	(10.5; 19.9)	(8.1; 11.8)	(6.2; 18.5)	(10.4; 16.1)	(6.8; 21.3)	(6.8; 12.4)	(7.2; 22.3)	(8.3; 14.5)
Burnout	83.1^a^	74.6	76.2	75.0	77.8	73.1	81.8	73.6
	(77.6; 87.5)	(71.8; 77.2)	(67.1; 83.5)	(71.1; 78.5)	(67.6; 85.5)	(68.6; 77.2)	(71.8; 88.8)	(69.0; 77.7)
Stress	82.2	78.1	84.2	77.8	74.1	74.3	75.3	72.1
	(76.6; 86.7)	(75.4; 80.6)	(75.8; 90.0)	(74.1; 81.1)	(63.6; 82.4)	(69.9; 78.3)	(64.6; 83.6)	(67.4; 76.3)
Unwanted sexual attention	11.0^a^	6.0	8.9	4.5	4.9	2.9	7.8	5.1
	(7.5; 15.8)	(4.7; 7.7)	(4.8; 16.1)	(3.0; 6.6)	(1.9; 12.0)	(1.7; 5.0)	(3.6; 16.0)	(3.3; 7.8)
Threat of violence	19.6^a^	9.1	13.9^a^	6.8	16.0	8.7	18.2	10.8
	(14.9; 25.4)	(7.4; 11.0)	(8.4; 21.9)	(4.9; 9.2)	(9.6; 25.5)	(6.4; 11.8)	(11.1; 28.2)	(8.1; 14.2)
Physical violence	2.3	1.6	3.0^a^	0.6	3.7	1.0	3.9	1.8
	(1.0; 5.2)	(1.0; 2.6)	(1.0; 8.4)	(0.2; 1.6)	(1.3; 10.33)	(0.4; 2.5)	(1.3; 10.8)	(0.9; 3.7)
Bullying	15.1	15.9	15.8	12.2	13.6	10.7	14.3	12.3
	(10.9; 20.4)	(13.8; 18.3)	(10.0; 24.2)	(9.7; 15.2)	(7.8; 22.7)	(8.0; 14.0)	(8.2; 23.8)	(9.4; 15.9)

^a^ p-value<0,05.

**Figure 2.**
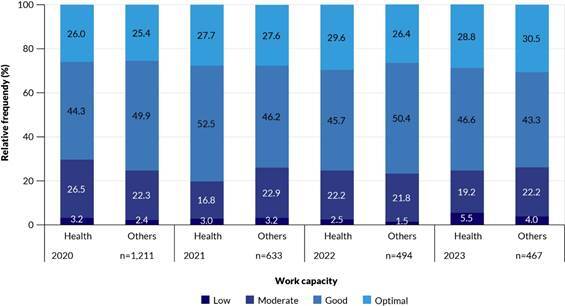
Proportion of workers in each work ability category over time, separately for health workers and other professionals. São Carlos, 2020-2023

Across all years assessed, most workers were classified as having good work ability. No statistically significant differences were found between the groups (p-value>0.050 in all years). Longitudinal analysis also showed no significant changes over time among health workers (p-value 0.131) or non-health-related workers (p-value 0.835) (Figure 2).

## Discussion

These findings revealed differences between health and non-health-related workers at the onset of the COVID-19 pandemic regarding psychosocial risk factors. Health professionals showed greater risks in emotional demands, self-rated health, burnout, unwanted sexual attention, and threats of violence. From 2022 onward, psychosocial risk factors among health workers were limited to emotional demands and threats of violence. Longitudinal analysis showed an increase in risks related to influence at work and predictability in this group. Non-health-related workers had higher psychosocial risks regarding influence at work, opportunities for development, meaning of work, work commitment, and clarity of roles and responsibilities. 

This study had some limitations. The main one was the decrease in the number of participants over time, possibly related to the absence of in-person contact between researchers and respondents, the time required to respond the questionnaires, and the saturation of online studies targeting this population during the pandemic. This reduction may have affected the accuracy of estimates and led to the underrepresentation of workers with limited internet access. Moreover, the predominance of remote work among non-health-related professionals may have been influenced by the study design itself, favoring the participation of individuals with higher digital literacy. It was not possible to determine whether health professionals worked on the front lines or in outpatient settings, which may influence the interpretation of the risks faced.

These findings reflect the ongoing emotional burden experienced by health professionals over the 36-month follow-up period. The literature supports this observation, particularly in high-pressure environments such as intensive care units, where work overload, emotional exhaustion, and long shifts are common ^14^. During the COVID-19 pandemic, new elements exacerbated this scenario: shortages of personal protective equipment, high risk of infection, family isolation, lack of clear information about the disease, difficulty accessing diagnostic tests, job dissatisfaction, and lack of social support ^5^ ^,^ ^15^ ^,^ ^16^. Health professionals, especially those on the front lines, showed higher rates of burnout and poorer general health perception compared to the general population ^9^ ^,^ ^17^ ^,^ ^18^.

Workplace violence also emerged as a critical factor. Between 2020 and 2022, health professionals were more exposed to threats and physical aggression, in addition to reports of unwanted sexual attention. Reported violence rates ranged from 42.0% to 45.0%, with verbal violence being the most common, especially among female respondents ^19^ ^,^ ^20^.

Despite the risks identified, the meaning attributed to work by health professionals may have served as a protective factor, linked to the perceived relevance of their roles ^21^. This lower psychosocial risk associated with meaning of work remained stable throughout the study. However, the growing perception of lack of influence at work and unpredictability in daily activities from 2021 onward indicated a progressive loss of control in response to demands imposed by the collapse of health systems. The lack of clear information about institutional changes heightened anxiety and a sense of helplessness ^9^ ^,^ ^15^ ^,^ ^16^.

Despite lower exposure to certain risk factors-such as opportunities for development and work commitment-the adverse context experienced during the COVID-19 pandemic was predominant. Comparing psychosocial factors during the pandemic to the pre-pandemic period also revealed both harmful and protective effects among nurses in Germany ^22^. Changes in the work environment under these circumstances increased psychosocial demands and affected mental stress levels in health workers, even in the presence of protective factors ^22^. The challenges faced by health workers due to inadequate and fragmented health systems contributed to their heightened vulnerability ^23^.

Conversely, non-health-related workers experienced greater psychosocial risks associated with opportunities for development, work commitment, role clarity, and meaning of work. The abrupt migration to working from home and the intensive use of technology contributed to this scenario ^24^. Remote workers reported social disconnection, lack of clear guidelines, technical difficulties, workload overload, and feelings of isolation. The literature links these factors to decreased engagement, loss of sense of community, and worsening mental health and well-being ^25^ ^,^ ^26^ ^,^ ^27^.

Other essential sectors, for example food services, building maintenance, transportation, security, and communication, also faced adverse conditions ^28^. Factors such as increased workloads, lack of training, conflicts with customers, social stigma, involuntary exposure to risk, and lack of social recognition were associated with higher occupational stress in these groups ^6^ ^,^ ^7^ ^,^ ^28^.

A longitudinal analysis showed that, although non-health-related professionals initially faced high risks related to influence at work, this risk gradually decreased-possibly linked to a greater sense of autonomy in remote work ^25^. Trust in leadership showed slight improvement in 2021, possibly supported by broader access to digital resources and platforms that became more established over time. Some of these changes, such as the use of technology and work flexibility, are expected to remain in the post-pandemic landscape ^29^.

Work ability remained satisfactory for both health and non-health-related professionals, with results similar to pre-pandemic levels. This suggests the resilience of Brazilian workers despite significant adversities ^30^. Still, health professionals experienced persistent psychosocial risks throughout the entire study period, highlighting the need for public policies and institutional strategies aimed at protecting and promoting workers’ mental health.

## References

[1] van der Molen HF, Nieuwenhuijsen K, Frings-Dresen MHW, de Groene G (2020). Work-related psychosocial risk factors for stress-related mental disorders: an updated systematic review and meta-analysis. BMJ Open.

[2] Andrade MA, Castro CSM, Batistão MV, Mininel VA, Sato TO (2022). Occupational profile, psychosocial aspects, and work ability of Brazilian workers during COVID-19 pandemic: IMPPAC cohort. Saf Health Work.

[3] Hunter JR, Meiring RM, Cripps A, Suppiah HT, Vicendese D, Kingsley MI (2021). Relationships between physical activity, work ability, absenteeism and presenteeism in Australian and New Zealand adults during COVID-19. Int J Environ Res Public Health.

[4] Aymerich C, Pedruzo B, Pérez JL, Laborda M, Herrero J, Blanco J (2022). COVID-19 pandemic effects on health worker’s mental health: systematic review and meta-analysis. Eur Psych.

[5] Hill JE, Harris C, Danielle LC, Boland P, Doherty AJ, Benedetto V (2022). The prevalence of mental health conditions in healthcare workers during and after a pandemic: systematic review and meta-analysis. J Adv Nurs.

[6] Lima LC, Alda JV (2024). Trabalhadoras essenciais abandonadas: psicodinâmica do trabalho e saúde mental de caixas de supermercado durante a pandemia de covid-19. Rev Bras Saude Ocup.

[7] Oliveira PTG, Junges JR (2023). Plataformas digitais de entrega de alimentação: condições de trabalho e riscos para a saúde. Saude Soc.

[8] Barros MBA, Lima MG, Malta DC, Szwarcwald CL, Azevedo RCS, Romero D (2020). Relato de tristeza/depressão, nervosismo/ansiedade e problemas de sono na população adulta brasileira durante a pandemia de COVID-19. Epidemiol Serv Saude.

[9] Lobo LAC, Rieth CE (2021). Saúde mental e Covid-19: uma revisão integrativa da literatura. Saude Debate.

[10] Serpa ALO, Pinto ALB, Diaz AP, Romano-Silva MA, Costa DS, Joaquim RM (2022). The mental health of Brazilian healthcare professionals during the COVID-19 pandemic: a longitudinal study. Braz J Psychiatry.

[11] Costa A, Caldas de Almeida T, Fialho M, Rasga C, Martiniano H, Santos O (2023). Mental health of healthcare professionals: two years of the covid-19 pandemic in Portugal. Int J Environ Res Public Health.

[12] Tuomi K, Ilmarinen J, Jahkola A, Katajarinne L, Tulkki A (2005). Índice de capacidade para o trabalho.

[13] Gonçalves JS, Moriguchi CS, Chaves TC, Sato TO (2021). Cross-cultural adaptation and psychometric properties of the short version of COPSOQ II-Brazil. Rev Saude Publica.

[14] Ramírez-Elvira S, Romero-Béjar JL, Suleiman-Martos N, Gómez-Urquiza JL, Monsalve-Reyes C, Cañadas-De la Fuente GA (2021). Prevalence, risk factors and burnout levels in intensive care unit nurses: a systematic review and meta-analysis. Int J Environ Res Public Health.

[15] Diogo PMJ, Sousa MOCLE, Rodrigues JRGDV, Silva TAAMAE, Santos MLF (2021). Emotional labor of nurses in the front line against the COVID-19 pandemic. Rev Bras Enferm.

[16] Da Rosa P, Brown R, Pravecek B, Carotta C, Garcia AS, Carson P (2021). Factors associated with nurses emotional distress during the COVID-19 pandemic. Appl Nurs Res.

[17] De Pasquale C, Conti D, Dinaro C, D’Antoni RA, La Delfa E, Di Nuovo S (2022). The COVID-19 pandemic and posttraumatic stress disorder: emotional impact on healthcare professions. Front Psychiatry.

[18] Swaminathan R, Mukundadura BP, Prasad S (2022). Impact of enhanced personal protective equipment on the physical and mental well-being of healthcare workers during COVID-19. Postgrad Med J.

[19] Hadavi M, Ghomian Z, Mohammadi F, Sahebi A (2023). Workplace violence against health care workers during the COVID-19 pandemic: a systematic review and meta-analysis. J Safety Res.

[20] Saragih ID, Tarihoran D, Rasool A, Saragih IS, Tzeng HM, Lin CJ (2022). Global prevalence of stigmatization and violence against healthcare workers during the COVID-19 pandemic: a systematic review and meta-analysis. J Nurs Scholarsh.

[21] Schmidt-Stiedenroth K, Mambrey V, Dreher A, Loerbroks A (2024). Psychosocial working conditions and mental health among medical assistants in Germany: a scoping review. BMC Public Health.

[22] Schulze S, Merz S, Thier A, Tallarek M, König F, Uhlenbrock G (2022). Psychosocial burden in nurses working in nursing homes during the COVID-19 pandemic: a cross-sectional study with quantitative and qualitative data. BMC Health Serv Res.

[23] Muñoz-Ortega S, Santamaría-Guayaquil D, Pluas-Borja J, Alvarado-Villa G, Sandoval V, Alvarado R (2024). Mental health in healthcare workers post-COVID-19: a latin american review and insights into personalized management strategies. J Pers Med.

[24] Liu W, Xu Y, Ma D (2021). Work-related mental health under COVID-19 restrictions: a mini literature review. Front Public Health.

[25] De Vincenzi C, Pansini M, Ferrara B, Buonomo I, Benevene P (2022). Consequences of COVID-19 on employees in remote working: challenges, risks and opportunities an evidence-based literature review.. Int J Environ Res Public Health.

[26] Bahamondes-Rosado ME, Cerdá-Suárez LM, Dodero Ortiz de Zevallos GF, Espinosa-Cristia JF (2023). Technostress at work during the COVID-19 lockdown phase (2020-2021): a systematic review of the literature. Front Psychol.

[27] Tobia L, Vittorini P, Di Battista G, D’Onofrio S, Mastrangeli G, Di Benedetto P (2024). Study on psychological stress perceived among employees in an italian university during mandatory and voluntary remote working during and after the COVID-19 pandemic. Int J Environ Res Public Health.

[28] Chowdhury N, Kainth A, Godlu A, Farinas HA, Sikdar S, Turin TC (2022). Mental health and well-being needs among non-health essential workers during recent epidemics and pandemics. Int J Environ Res Public Health.

[29] Tønnessen Ø, Dhir A, Flåten BT (2021). Digital knowledge sharing and creative performance: work from home during the COVID-19 pandemic. Technol Forecast Soc Change.

[30] Martinez MC, Latorre MRDO, Fischer FM (2016). Testando o modelo da casa da capacidade para o trabalho entre profissionais do setor hospitalar. Rev Bras Epidemiol.

