# Supplementary material for: Psychosocial risks and work ability among health workers throughout the COVID-19 pandemic: a longitudinal follow-up study, Brazil, 2020-2023
Source: Epidemiol Serv Saude. 2026 Mar 9;35:e20240798. doi: 10.1590/S2237-96222026v35e20240798.en (PMC12970836; doi:10.1590/S2237-96222026v35e20240798.en)
Supplement: Translation

# Translation: Riscos psicossociais e capacidade para o trabalho em trabalhadores da saúde ao longo da pandemia de covid-19: estudo longitudinal com acompanhamento, Brasil, 2020-2023

**DOI:** 10.1590/S2237-96222026v35e20240798.pt

**Authors:** Carolina Luciane Nogueira Martinez, Cristiane Shinohara Moriguchi, Marcela Alves Andrade, Tatiana de Oliveira Sato

**Affiliations:** 1 Universidade Federal de São Carlos, Centro de Ciências Biológicas e da Saúde, Programa de Pós-Graduação em Fisioterapia, São Carlos, SP, Brasil; 2 Universidade Federal de São Carlos, Centro de Ciências Biológicas e da Saúde, Departamento de Fisioterapia, São Carlos, SP, Brasil

**Keywords:** SARS-CoV-2, Riscos Ocupacionais, Impacto Psicossocial, Avaliação da Capacidade de Trabalho, Estudos Longitudinais

## Abstract

**Objetivo:**

Comparar profissionais da saúde e profissionais não relacionados à assistência em saúde quanto aos riscos psicossociais e à capacidade para o trabalho durante 36 meses de acompanhamento.

**Métodos:**

Tratou-se de estudo longitudinal prospectivo. Os participantes responderam a um questionário sociodemográfico, ao Copenhagen Psychosocial Questionnaire II e ao índice da capacidade para o trabalho. Os dados foram analisados de forma descritiva pelas frequências absoluta e relativa e intervalo de confiança de 95% (IC95%). Testes qui-quadrado e Cochran foram usados para a comparação inter e intragrupos.

**Resultados:**

Participaram 1.211 trabalhadores (219 profissionais da saúde e 992 profissionais não relacionados à assistência em saúde) em 2020. Nesse ano, três a cada quatro profissionais de saúde enfrentaram altas demandas emocionais (75,8%; IC95% 69,7; 81,0%), percepção ruim de saúde (14,6%; IC95% 10,5; 19,9%), burnout (83,1%; IC95% 77,6; 87,5%), atenção sexual indesejada (11,0%; IC95% 7,5; 15,8%), ameaças de violência (19,6%; IC95% 14,9; 25,4%) e violência física (2,3%; IC95% 1,0; 5,2%). Em 2023, as demandas emocionais (71,4%; IC95% 60,5; 80,3%) e as ameaças de violência (18,2%; IC95% 11,1; 28,2%) permaneceram maiores. A capacidade para o trabalho manteve-se estável em ambos os grupos. A frequência da classificação foi boa em 2020 (44,3 e 49,9), 2021 (52,5 e 46,2), 2022 (45,7 e 50,4) e 2023 (46,6 e 43,3) nos profissionais da saúde e nos profissionais não relacionados à assistência em saúde.

**Conclusões:**

Os profissionais de saúde estiveram expostos a riscos psicossociais durante a pandemia, principalmente a demandas emocionais e ameaças de violência, o que indica necessidade de intervenção no ambiente de trabalho.

Aspectos éticos

Esta pesquisa respeitou os princípios éticos, obtendo os seguintes dados de aprovação:

Comitê de ética em pesquisa: Universidade Federal de São Carlos

Número do parecer: 4.166.321

Data de aprovação: 21/7/2020

Certificado de apresentação de apreciação ética: 31885020.9.0000.5504

Registro do consentimento livre e esclarecido: Obtido de todos os participantes antes da coleta.

## Introdução

Os riscos psicossociais relacionados ao trabalho resultam da interação entre fatores do ambiente de trabalho com os fatores humanos, podendo influenciar a saúde, o desempenho e a capacidade para o trabalho ^1^. A pandemia de covid-19 modificou as condições de trabalho sendo necessário acompanhar seus efeitos nos riscos psicossociais e na capacidade para o trabalho ^2^ ^,^ ^3^.

Os profissionais de saúde enfrentaram uma variedade de desafios nesse período. As alterações no ambiente de trabalho, como aumento da carga de trabalho, demandas conflitantes, falta de suporte social, longas jornadas e rotação de turnos, contribuíram para o aumento dos fatores de risco desses profissionais ^4^ ^,^ ^5^. A pandemia teve efeitos diretos nos profissionais da saúde, a exemplo do aumento nos índices de ansiedade, depressão, insônia, estresse pós-traumático e burnout ^5^. 

A rotina exaustiva de trabalho também esteve presente em outras áreas. Trabalhadores considerados essenciais, como caixas de supermercados e entregadores, apresentaram exaustão emocional, risco de contaminação e precarização do trabalho durante o período pandêmico no Brasil ^6^ ^,^ ^7^. De maneira geral, a pandemia afetou a saúde mental da população adulta, com prevalência de 52,6% de sintomas de ansiedade em levantamento com 45 mil brasileiros em 2020 ^8^. No entanto, os profissionais de saúde apresentaram maior prevalência de depressão, estresse agudo e burnout em relação à população geral ^4^ ^,^ ^9^. Sugere-se que os fatores de risco psicossociais a que esses profissionais estiveram expostos durante a pandemia também foram maiores que os vivenciados pela população em geral. 

Um estudo longitudinal com acompanhamento de seis meses em trabalhadores de saúde brasileiros demonstrou piora da saúde mental durante o primeiro ano da pandemia ^10^. Em Portugal, as alterações da saúde mental dos profissionais de saúde foram acompanhadas durante dois anos, e encontraram-se a persistência de sintomas de burnout e a redução de sintomas de ansiedade e estresse pós-traumático no período 2020-2021 ^11^. 

A capacidade para o trabalho também está relacionada às restrições sociais e às alterações no trabalho durante a pandemia ^3^. A capacidade para o trabalho é resultado do equilíbrio entre os recursos mentais e físicos dos trabalhadores e as demandas do trabalho e pode ser avaliada por meio do índice de capacidade para o trabalho ^12^.

Este estudo teve como objetivo comparar os profissionais da saúde com profissionais não relacionados à assistência em saúde quanto aos riscos psicossociais e à capacidade para o trabalho durante 36 meses de acompanhamento após o início da pandemia de covid-19 no Brasil. Embora o adoecimento de profissionais da saúde seja amplamente estudado, este estudo avançou na identificação de fatores de risco psicossociais e no acompanhamento longitudinal.

Métodos

Delineamento do estudo

Tratou-se de estudo longitudinal de coorte com seguimento prospectivo de 36 meses. Os dados foram obtidos a cada 12 meses em 2020 (linha de base), 2021, 2022 e 2023. 

Contexto

O estudo foi realizado de forma on-line, divulgado em anúncios na imprensa local, mídias sociais e convites por e-mail. Tratou-se de estudo com abrangência nacional, divulgado pela Coordenadoria de Comunicação Social da universidade, que se difundiu em outros órgãos de imprensa (sítios eletrônicos, rádios e canais de televisão). As mídias sociais foram institucionais, assim como os e-mails dos quais os convites foram enviados. Os e-mails foram obtidos em sítios eletrônicos públicos e enviados à rede de contato dos pesquisadores. 

Participantes

Este estudo foi vinculado ao projeto “Implicações da pandemia de covid-19 nos aspectos psicossociais e capacidade para o trabalho entre trabalhadores brasileiros”, que avalia anualmente trabalhadores brasileiros desde 2020 quanto aos fatores psicossociais e à capacidade para o trabalho ^2^.

Variáveis

Os participantes responderam ao questionário sociodemográfico, ao índice da capacidade para o trabalho ^12^ e à versão curta do Copenhagen Psychosocial Questionnaire II (COPSOQ II-Br) ^13^.

Fonte de dados

Foram incluídos trabalhadores que aceitaram participar do estudo com idade igual ou superior a 18 anos, residentes no Brasil e atuantes em qualquer setor profissional. Não foram incluídos estudantes, estagiários, aposentados e indivíduos que apresentaram dados repetidos. Os profissionais de saúde compreenderam enfermeiros, fisioterapeutas, farmacêuticos, fonoaudiólogos, médicos, médicos veterinários, nutricionistas, educadores físicos, psicólogos, técnicos de enfermagem, administrativos do setor saúde e professores de cursos de saúde.

Na avaliação inicial, os trabalhadores responderam a um questionário sociodemográfico e ocupacional, versão curta do COPSOQ II-Br e do índice da capacidade para o trabalho. Nas avaliações anuais subsequentes, os trabalhadores foram contatados por e-mail e convidados a responderem o COPSOQ II-Br e o índice da capacidade para o trabalho. As avaliações foram realizadas entre junho e setembro de cada ano (2020-2023) e totalizaram quatro avaliações.

O questionário sociodemográfico e ocupacional continha questões relacionadas a sexo, idade, raça/cor da pele, cidade, estado, estado civil, número de filhos, peso, altura, fumante ou não, uso de medicamento, escolaridade, profissão, tempo de profissão, tipo de vínculo do trabalho atual, setor profissional, renda familiar, se estava empregado, se perdeu emprego na pandemia e questões sobre a pandemia de covid-19 (por exemplo: contraiu covid-19, recebeu vacinas, usou medidas de proteção individual etc.). 

O COPSOQ II-Br foi usado para identificar os riscos psicossociais no trabalho ^13^. Esse instrumento adotou abordagem multifacetada, abrangendo 23 dimensões e 40 questões ^14^. O COPSOQ II-Br identificou os seguintes fatores de risco: demandas quantitativas, ritmo de trabalho, demandas emocionais, possibilidades de desenvolvimento, significado do trabalho, comprometimento, reconhecimento, suporte social, satisfação no trabalho, conflitos família e trabalho, burnout, estresse e comportamentos ofensivos (atenção sexual indesejada, ameaças de violência, violência física e bullying) ^13^. O COPSOQ II-Br foi traduzido e adaptado transculturalmente para a população brasileira ^13^.

As questões foram pontuadas de acordo com uma escala com cinco opções de resposta. Apenas a questão 1B apresentou pontuação invertida. A pontuação foi feita pela soma das questões de cada dimensão, exceto para a dimensão comportamentos ofensivos. Quanto maior a pontuação, maior o risco identificado.

O índice da capacidade para o trabalho, traduzido para o português do Brasil, avaliou a percepção do trabalhador sobre sua capacidade de trabalho, considerando a saúde e as demandas do trabalho ^12^. Esse índice não requereu avaliador para sua aplicação e foi composto por sete dimensões: capacidade para o trabalho comparada com a melhor de toda vida; capacidade para o trabalho em relação a exigências do trabalho; número de doenças atuais diagnosticadas pelo médico; perda estimada para o trabalho por causa de doenças; faltas ao trabalho por doenças nos últimos 12 meses; prognóstico próprio da capacidade para o trabalho daqui a dois anos; e recursos mentais. 

O escore do índice da capacidade para o trabalho variou de 7 a 49 pontos, e a capacidade para o trabalho foi classificada em: baixa (pontuação de 7 a 27), moderada (pontuação de 28 a 36), boa (pontuação de 37 a 43) e ótima (pontuação de 44 a 49) ^12^.

Viés

A estratégia de divulgação da pesquisa e o meio de contato com os participantes podem ter introduzido viés de seleção, uma vez que indivíduos com menor acesso à internet e menor familiaridade com o meio digital foram sub-representados na amostra.

Tamanho do estudo

A partir da divulgação do estudo, todos os participantes da pesquisa que atendessem aos critérios de inclusão foram considerados para a análise, não havendo balanceamento dos grupos.

Métodos estatísticos

A caracterização do perfil sociodemográfico, dos riscos psicossociais e da capacidade para o trabalho foi realizada por meio de frequências absoluta e relativa e intervalo de confiança de 95% (IC95%) para as variáveis categóricas. Média, desvio-padrão, mínimo e máximo foram calculados para as variáveis contínuas. A comparação entre o grupo de profissionais de saúde e o de profissionais não relacionados à assistência em saúde foi realizada por meio do teste qui-quadrado. A comparação entre os períodos de avaliação, separadamente para cada grupo, foi realizada por meio do teste de Cochran. O nível de significância foi 5%.

## Resultados

Foram incluídos 1.211 trabalhadores em 2020, sendo 219 profissionais da saúde e 992 profissionais não relacionados à assistência em saúde (Figura 1). A idade média dos trabalhadores em 2020 foi 37,7 anos (desvio-padrão 10,5), sendo semelhante (p-valor 0,125) entre os profissionais da saúde (média 36,7; desvio-padrão 9,5) e aqueles não relacionados à assistência em saúde (média 37,9; desvio-padrão 10,8). O grupo de profissionais da saúde apresentou maior proporção de mulheres (75,8%), nível universitário (88,1%), menor proporção em teletrabalho (42,9%), maior proporção de infecção por coronavírus (10,5%) e maior proporção de profissionais com renda entre 3 e 12 salários mínimos (67,6%) (Tabela 1). As demais variáveis foram similares entre os grupos. 

Foram identificadas diferenças significativas entre os grupos quanto aos riscos psicossociais (Tabela 2). Os profissionais de saúde apresentaram maior risco para as demandas emocionais em todos os momentos avaliados (p-valor<0,050). Para as dimensões percepção geral de saúde, burnout, atenção sexual indesejada, ameaça de violência e violência física, houve diferença entre os grupos, com maior risco nos profissionais da saúde em 2020 e 2021.

Nos profissionais não relacionados à assistência em saúde, foram encontrados maiores riscos nas dimensões influência no trabalho em 2020 e 2023, possibilidade de desenvolvimento em 2020, 2021 e 2022, significado do trabalho em 2020, 2021, 2022 e 2023, comprometimento com o trabalho em 2020 e 2021 e clareza de papéis em 2022.

**Figura 1.** Número de participantes durante o seguimento do estudo. São Carlos, 2020-2023 (n=1.211)

**Tabela 1:** Frequência relativa (%) e intervalo de confiança de 95% (IC95%) das características sociodemográficas e ocupacionais na linha de base de acordo com os grupos de profissionais da saúde (n=219) e profissionais não relacionados à assistência em saúde (não saúde) (n=992). São Carlos, 2020 (n=1.211)

Característica	Total	Profissionais de saúde (n=219)	Profissionais não saúde (n=992)	p-valor
	(n=1.211)	% (IC95%)	% (IC95%)	
	% (IC95%)			
Sexo				
Feminino	51,9 (49,1; 54,7)	75,8 (69,7; 81,0)	46,7 (43,6; 49,8)	<0,001
Masculino	48,1 (45,3; 50,9)	24,2 (19,0; 30,3)	53,3 (50,2; 56,4)	
Situação conjugal				0,664
Solteiro/viúvo/divorciado	45,3 (42,5; 48,1)	46,6 (40,1; 53,2)	45,0 (41,9; 48,1)	
Casado/união estável	54,7 (51,9; 57,5)	53,4 (46,8; 59,9)	55,0 (51,9; 58,1)	
Escolaridade				0,03
Universitário	83,2 (80,9; 85,1)	88,1 (83,2; 91,8)	82,1 (79,5; 84,3)	
Não universitário	16,8 (14,8; 19,1)	11,9 (8,2; 16,8)	17,9 (15,7; 20,4)	
Fumante	5,8 (4,6; 7,2)	5,5 (3,2; 9,3)	5,8 (4,5; 7,5)	0,582
Contrato de trabalho				0,247
Servidor público	41,0 (38,2; 43,7)	39,3 (33,0; 45,9)	41,4 (38,3; 44,4)	
Serviço privado	44,0 (41,2; 46,8)	41,1 (34,8; 47,7)	44,7 (41,6; 47,8)	
Informal	0,2 (0,0; 0,6)	0,5 (0,1; 2,5)	0,5 (0,0; 0,6)	
Autônomo	0,8 (0,4; 1,5)	0,9 (0,2; 3,3)	0,8 (0,4; 1,6)	
Empresário	0,7 (0,4; 1,4)	0,5 (0,1; 2,5)	0,8 (0,4; 1,6)	
Setor profissional				<0,001
Saúde	18,1 (16,0; 20,3)	100 (98,3; 100,0)	-	
Educação	37,2 (34,5; 39,9)	-	45,4 (42,3; 48,5)	
Comércio essencial	3,3 (2,4; 4,5)	-	4,0 (3,0; 5,4)	
Comércio não essencial	5,9 (4,7; 7,3)	-	7,2 (5,7; 8,9)	
Construção civil	2,1 (1,5; 3,1)	-	2,6 (1,8; 3,8)	
Segurança pública	1,3 (0,8; 2,4)	-	1,6 (1,0; 2,3)	
Transporte público	0,3 (0,1; 0,8)	-	0,4 (0,2; 1,0)	
Agricultura	1,2 (0,7; 2,0)	-	1,5 (0,9; 2,5)	
Transporte privado	0,3 (0,1; 0,8)	-	0,4 (0,2; 1,0)	
Delivery	0,2 (0,0; 0,6)	-	0,2 (0,1; 0,7)	
Limpeza	0,1 (0,0; 0,5)	-	0,1 (0,0; 0,6)	
Serviço de logística	4,0 (3,0; 5,2)	-	4,8 (3,7; 6,4)	
Indústria	6,8 (5,5; 8,3)	-	8,3 (6,7; 10,1)	
Serviço público	5,3 (4,2; 6,7)	-	6,5 (5,1; 8,1)	
Organizações não governamentais	0,3 (0,1; 0,8)	-	0,4 (0,2; 1,0)	
Serviço bancário	1,5 (0,9; 2,3)	-	1,8 (1,1; 2,8)	
Informática	7,8 (5,1; 7,9)	-	7,8 (6,3; 9,6)	
Advocacia	3,6 (2,6; 4,7)	-	4,3 (3,2; 5,8)	
Comunicação	1,8 (1,2; 2,7)	-	2,2 (1,5; 3,3)	
Turismo e hotel	0,4 (0,2; 1,0)	-	0,4 (0,2; 1,1)	
Teletrabalho	74,1 (71,5; 76,5)	42,9 (36,5; 49,5)	80,9 (78,4; 83,3)	<0,001
Infecção por coronavírus	5,4 (4,2; 6,8)	10,5 (7,1; 15,3)	4,2 (3,1; 5,7)	<0,001
Raça/cor da pele				0,556
Branca	49,3 (46,5; 52,1)	42,9 (36,5; 49,5)	50,7 (47,6; 53,8)	
Não branca	5,7 (2,1; 4,1)	3,2 (1,6; 6,4)	5,5 (2,0; 4,1)	
Uso de medicamentos	40,4 (37,5; 43,0)	41,1 (34,8; 47,7)	40,2 (37,0; 43,1)	0,769
Renda familiar mensal (salários mínimos)				0,017
Não declarada	5,0 (3,9; 6,3)	2,3 (1,0; 5,2)	5,5 (4,3; 7,1)	
Até 1	1,7 (1,1; 2,5)	1,4 (0,5; 3,9)	1,7 (1,1; 2,7)	
01/mar	18,0 (15,9; 20,3)	15,5 (11,3; 20,9)	18,5 (16,2; 21,1)	
03/jun	26,6 (24,2; 29,1)	31,5 (25,7; 37,9)	25,5 (22,9; 28,3)	
06/set	16,0 (14,1; 18,2)	21,0 (16,1; 26,9)	14,9 (12,8; 17,3)	
09/dez	14,4 (12,5; 16,5)	15,1 (10,9; 20,4)	14,2 (12,2; 16,5)	
>12	18,4 (16,3; 20,7)	13,2 (9,4; 18,4)	14,2 (17,2; 22,1)	
Índice de massa corporal				0,614
Abaixo do peso	1,5 (0,9; 2,3)	1,8 (0,7; 4,6)	1,4 (0,8; 2,3)	
Normal	39,2 (36,5; 42,0)	42,0 (35,7; 48,6)	38,6 (35,6; 41,7)	
Sobrepeso	35,7 (33,0; 38,4)	35,6 (29,6; 42,1)	35,7 (32,8; 38,7)	
Obeso	23,6 (21,3; 26,1)	20,5 (15,7; 26,4)	24,3 (21,7; 27,1)	

Foram identificadas diferenças significativas entre os grupos quanto aos riscos psicossociais (Tabela 2). Os profissionais de saúde apresentaram maior risco para as demandas emocionais em todos os momentos avaliados (p-valor<0,050). Para as dimensões percepção geral de saúde, burnout, atenção sexual indesejada, ameaça de violência e violência física, houve diferença entre os grupos, com maior risco nos profissionais da saúde em 2020 e 2021.

Nos profissionais não relacionados à assistência em saúde, foram encontrados maiores riscos nas dimensões influência no trabalho em 2020 e 2023, possibilidade de desenvolvimento em 2020, 2021 e 2022, significado do trabalho em 2020, 2021, 2022 e 2023, comprometimento com o trabalho em 2020 e 2021 e clareza de papéis em 2022.

Foram identificadas diferenças significativas para a dimensão influência no trabalho ao longo dos anos, com aumento do risco nos profissionais de saúde ao longo de 2020 e 2022 e redução em 2023, sendo significativas as diferenças entre 2020-2022 (p-valor 0,040), 2021-2023 (p-valor 0,040) e 2022-2023 (p-valor 0,003). Na pandemia, também houve aumento do fator de risco previsibilidade em 2021 (p-valor 0,010), 2022 (p-valor 0,022) e 2023 (p-valor 0,022) quando comparado a 2020. 

Para os profissionais não relacionados à assistência em saúde, foi identificada diminuição do risco na influência do trabalho na comparação de 2020-2022 (p-valor 0,005) e 2020-2023 (p-valor 0,018) e no fator confiança na gestão (p-valor 0,004) na comparação 2021-2023. Por outro lado, esses profissionais apresentaram aumento do risco no significado do trabalho em 2020 para 2021 (p-valor 0,001), 2022 (p-valor 0,020) e 2023 (p-valor 0,001). 

**Tabela 2:** Frequência relativa (%) e intervalo de confiança de 95% (IC95%) dos aspectos psicossociais relacionados ao trabalho entre profissionais de saúde (saúde) e profissionais não relacionados à assistência em saúde (não saúde). São Carlos, 2020-2023 (n=1.211)

Dimensões	2020		2021		2022		2023	
	Saúde (n=219)	Não saúde (n=992)	Saúde (n=101)	Não saúde (n=532)	Saúde (n=81)	Não saúde (n=413)	Saúde (n=77)	Não saúde (n=390)
	% (IC95%)	% (IC95%)	% (IC95%)	% (IC95%)	% (IC95%)	% (IC95%)	% (IC95%)	% (IC95%)
Demandas quantitativas	9,6	9,7	10,9	12,4	18,5	13,3	10,4	14,9
	(6,4; 14,2)	(8,0; 11,7)	(6,2; 8,5)	(9,9; 15,5)	(11,6; 28,3)	(10,4; 16,9)	(5,4; 19,2)	(11,7; 18,7)
Ritmo de trabalho	51,6	48,6	49,5	46,8	49,4	42,6	50,6	45,1
	(45,0; 58,1)	(45,5; 51,7)	(39,9; 59,1)	(42,6; 51,0)	(38,8; 60,0)	(37,9; 47,4)	(39,7; 61,5)	(40,3; 50,0)
Demandas emocionais	75,8^a^	48,8	75,2^a^	43,2	67,9^a^	48,2	71,4 ^a^	49,7
	(69,7; 81,0)	(45,7; 51,9)	(66,0; 82,6)	(39,1; 47,5)	(57,1; 77,1)	(43,4; 53,0)	(60,5; 80,3)	(44,8; 54,7)
Influência no trabalho	25,6	36,1 ^a^	27,7	33,1	29,6	28,6	16,9	29,7 ^a^
	(20,2; 31,7)	(33,2; 39,1)	(19,9; 37,1)	(29,2; 37,2)	(20,8; 40,3)	(24,4; 33,1)	(10,1; 26,8)	(25,4; 34,5)
Possibilidade de desenvolvimento	2,3	8,6 ^a^	2	7,3 ^a^	-	7,5 ^a^	2,6	8,5
	(1,0; 5,2)	(7,0; 10,5)	(0,5; 6,9)	(5,4; 9,9)		(5,3; 10,5)	(0,7; 9,0)	(6,1; 11,6)
Significado do trabalho	6,8	12,2 ^a^	6,9	16,7 ^a^	4,9	14,8 ^a^	6,5	14,9 ^a^
	(4,2; 11,0)	(10,3; 14,4)	(3,4; 13,6)	(13,8; 20,1)	(1,9; 12,0)	(11,7; 18,5)	(2,8; 14,3)	(11,7; 18,7)
Comprometimento com	6,8	12,2 ^a^	5,9	13,9 ^a^	7,4	14,5	13	13,8
o trabalho	(4,2; 11,0)	(10,3; 14,4)	(2,7; 12,4)	(11,2; 17,1)	(3,4; 15,2)	(11,5; 18,2)	(7,2; 22,3)	(10,8; 17,6)
Previsibilidade	24,7	26,1	27,7	26,5	22,2	24,9	27,3	24,1
	(19,4; 30,8)	(23,5; 28,9)	(19,9; 37,1)	(22,9; 30,4)	(14,5; 32,4)	(21,0; 29,3)	(18,6; 38,1)	(20,1; 28,6)
Reconhecimento do trabalho	19,2	17,9	15,8	16,9	16	18,6	19,5	16,4
	(14,5; 24,9)	(15,7; 20,4)	(10,0; 24,2)	(14,0; 20,3)	(9,6; 25,5)	(15,1; 22,7)	(12,1; 29,7)	(13,1; 20,4)
Clareza de papéis	11,4	10,6	10,9	12,6	4,9	14,3 ^a^	15,6	13,1
	(7,8; 16,3)	(8,8; 12,6)	(6,2; 18,5)	(10,0; 15,7)	(1,9; 12,0)	(11,2; 18,0)	(9,1; 25,3)	(10,1; 16,8)
Qualidade de liderança	28,8	26,2	25,7	23,1	21	25,4	32,5	26,2
	(23,2; 35,1)	(23,6; 29,0)	(18,2; 35,0)	(19,7; 26,9)	(13,5; 31,1)	(21,5; 29,8)	(23,1; 43,5)	(22,0; 30,7)
Apoio social dos supervisores	19,6	21,1	17,8	18,4	17,3	20,6	23,4	21,8
	(14,9; 25,4)	(18,6; 23,7)	(11,6; 26,4)	(15,4; 21,9)	(10,6; 26,9)	(17,0; 24,7)	(15,3; 34,0)	(18,0; 26,1)
Satisfação com trabalho	17,4	17,9	11,9	18,8	16	17,9	24,7	18,7
	(12,9; 22,9)	(15,7; 20,4)	(6,9; 19,6)	(15,7; 22,3)	(9,6; 25,5)	(14,5; 21,9)	(16,4; 35,3)	(15,1; 22,9)
Conflito entre trabalho e família	47	46,1	45,5	47	40,7	45	45,5	49,7
	(40,5; 53,6)	(43,0; 49,2)	(36,2; 55,2)	(42,8; 51,2)	(30,7; 51,6)	(40,3; 49,9)	(34,8; 56,5)	(44,8; 54,7)
Confiança na gestão	9,6	9,5	10,9	7	6,2	10,4	15,6	11,5
	(6,4; 14,2)	(7,8; 11,5)	(6,2; 18,5)	(5,1; 9,4)	(2,7; 13,6)	(7,8; 13,7)	(9,1; 25,3)	(8,7; 15,1)
Justiça e respeito	23,3	19,7	18,8	19,2	19,8	20,3	28,6	21,8
	(18,2; 29,3)	(17,3; 22,2)	(12,4; 27,5)	(16,0; 22,7)	(12,5; 29,7)	(16,7; 24,5)	(19,7; 39,5)	(18,0; 26,1)
Percepção geral de saúde	14,6 ^a^	9,8	10,9	13	12,3	9,2	13	11
	(10,5; 19,9)	(8,1; 11,8)	(6,2; 18,5)	(10,4; 16,1)	(6,8; 21,3)	(6,8; 12,4)	(7,2; 22,3)	(8,3; 14,5)
Burnout	83,1^a^	74,6	76,2	75	77,8	73,1	81,8	73,6
	(77,6; 87,5)	(71,8; 77,2)	(67,1; 83,5)	(71,1; 78,5)	(67,6; 85,5)	(68,6; 77,2)	(71,8; 88,8)	(69,0; 77,7)
Estresse	82,2	78,1	84,2	77,8	74,1	74,3	75,3	72,1
	(76,6; 86,7)	(75,4; 80,6)	(75,8; 90,0)	(74,1; 81,1)	(63,6; 82,4)	(69,9; 78,3)	(64,6; 83,6)	(67,4; 76,3)
Atenção sexual indesejada	11,0^a^	6	8,9	4,5	4,9	2,9	7,8	5,1
	(7,5; 15,8)	(4,7; 7,7)	(4,8; 16,1)	(3,0; 6,6)	(1,9; 12,0)	(1,7; 5,0)	(3,6; 16,0)	(3,3; 7,8)
Ameaça de violência	19,6^a^	9,1	13,9^a^	6,8	16	8,7	18,2	10,8
	(14,9; 25,4)	(7,4; 11,0)	(8,4; 21,9)	(4,9; 9,2)	(9,6; 25,5)	(6,4; 11,8)	(11,1; 28,2)	(8,1; 14,2)
Violência física	2,3	1,6	3,0^a^	0,6	3,7	1	3,9	1,8
	(1,0; 5,2)	(1,0; 2,6)	(1,0; 8,4)	(0,2; 1,6)	(1,3; 10,33)	(0,4; 2,5)	(1,3; 10,8)	(0,9; 3,7)
Bullying	15,1	15,9	15,8	12,2	13,6	10,7	14,3	12,3
	(10,9; 20,4)	(13,8; 18,3)	(10,0; 24,2)	(9,7; 15,2)	(7,8; 22,7)	(8,0; 14,0)	(8,2; 23,8)	(9,4; 15,9)

^a^ p-valor <0,05.

A maioria dos trabalhadores se enquadrou na categoria boa capacidade para o trabalho em todos os anos avaliados. Não houve diferença significante entre os grupos (p-valor>0,050 em todos os anos). A análise ao longo do tempo também mostrou que não houve mudanças significativas entre profissionais de saúde (p-valor=0,131) e aqueles não relacionados à assistência em saúde (p-valor=0,835) ao longo do tempo (Figura 2).

## Discussão

Estes achados revelaram diferenças entre profissionais de saúde e aqueles não relacionados à assistência em saúde no início da pandemia de covid-19 em relação aos fatores de risco psicossociais. Os profissionais de saúde apresentaram maiores riscos quanto a demandas emocionais, saúde geral, burnout, atenção sexual indesejada e ameaças de violência. A partir de 2022, os fatores de risco psicossociais se restringiram a demandas emocionais e ameaças de violência nos profissionais de saúde. A análise longitudinal revelou aumento dos riscos relacionados à influência no trabalho e à previsibilidade nos profissionais da saúde. Os profissionais não relacionados à assistência em saúde apresentaram maior risco psicossocial para influência no trabalho, possibilidade de desenvolvimento, significado do trabalho, comprometimento no trabalho e clareza de papéis. 

Este estudo apresentou algumas limitações. A principal delas foi a diminuição do número de participantes ao longo do tempo, possivelmente relacionada à ausência de contato presencial entre pesquisadores e respondentes, ao tempo necessário para preencher os questionários e ao excesso de estudos on-line voltados a essa população durante a pandemia. Essa redução pode ter afetado as estimativas dos resultados e gerado sub-representação de trabalhadores com menor acesso à internet. Ademais, a predominância de trabalhadores em regime remoto entre os profissionais não vinculados à saúde pode estar relacionada ao próprio desenho do estudo, o que favorece a participação de indivíduos com maior letramento digital. Não foi possível determinar se os profissionais de saúde atuavam na linha de frente ou em serviços ambulatoriais, o que pode influenciar a interpretação dos riscos enfrentados.

**Figura 2:** Proporção de trabalhadores em cada categoria de capacidade para o trabalho ao longo do tempo, separadamente para os grupos de profissionais da saúde e de outros profissionais. São Carlos, 2020-2023

Estes achados refletiram o impacto emocional contínuo enfrentado pelos profissionais de saúde ao longo dos 36 meses de acompanhamento. A literatura corrobora esse achado, especialmente em ambientes de alta pressão como unidades de terapia intensiva, onde há sobrecarga de trabalho, exaustão emocional e jornadas extensas ^14^. Durante a pandemia, novos elementos agravaram esse cenário: escassez de equipamentos de proteção individual, risco elevado de contaminação, isolamento familiar, falta de informações claras sobre a doença, dificuldade de acesso a testes diagnósticos, insatisfação no trabalho e ausência de suporte social ^5^ ^,^ ^15^ ^,^ ^16^. Os profissionais da saúde, especialmente os que atuaram na linha de frente, apresentaram maior incidência de burnout e pior percepção de saúde geral em comparação com a população geral ^9^ ^,^ ^17^ ^,^ ^18^.

A violência no ambiente de trabalho também se destacou como fator crítico. Entre 2020 e 2022, os profissionais de saúde foram mais expostos a ameaças e agressões físicas, além de relatos de atenção sexual indesejada. Foram identificadas taxas de violência que variaram entre 42,0% e 45,0%, sendo mais prevalente a violência verbal, principalmente entre as mulheres ^19^ ^,^ ^20^.

Apesar dos riscos identificados, o significado atribuído ao trabalho pelos profissionais de saúde pode ser considerado fator protetivo associado à percepção de relevância do próprio trabalho ^21^. Esse menor risco psicossocial relacionado ao significado do trabalho permaneceu estável ao longo do estudo. Contudo, o aumento na percepção de falta de influência no trabalho e de imprevisibilidade nas atividades a partir de 2021 indicou a crescente perda de controle diante das exigências impostas pelo colapso dos sistemas de saúde. A falta de informações claras sobre mudanças institucionais intensificou a ansiedade e a sensação de desamparo ^9^ ^,^ ^15^ ^,^ ^16^.

Apesar da menor exposição a alguns fatores de riscos em dimensões relacionadas à possibilidade de desenvolvimento e comprometimento no trabalho, a presença de contexto adverso vivenciado na pandemia foi preponderante. A comparação de fatores psicossociais durante a pandemia com o período pré-pandêmico também demonstrou efeitos negativos e protetivos durante a pandemia em enfermeiras na Alemanha ^22^. As mudanças no ambiente de trabalho durante esse cenário aumentaram as demandas psicossociais com influência no estresse mental dos trabalhadores da saúde, apesar da presença de fatores protetivos ^22^. As condições enfrentadas pelos profissionais de saúde decorrentes de sistemas de saúde inadequados e fragmentados contribuíram para a maior vulnerabilidade desses trabalhadores ^23^.

Em contrapartida, profissionais não vinculados à assistência à saúde apresentaram maior exposição a riscos psicossociais relacionados à possibilidade de desenvolvimento, comprometimento com o trabalho, clareza de papéis e significado do trabalho. A migração abrupta para o regime remoto e o uso intensivo de tecnologias contribuíram para esse cenário ^24^. Os trabalhadores remotos relataram desconexão social, falta de diretrizes claras, dificuldades técnicas, sobrecarga de funções e sensação de isolamento. A literatura associa esses fatores à deterioração do engajamento, à perda do senso de comunidade e à piora na saúde mental e no bem-estar ^25^ ^,^ ^26^ ^,^ ^27^.

Outros segmentos considerados essenciais, como os das áreas de alimentação, manutenção predial, transporte, segurança e comunicação, também enfrentaram condições adversas ^28^. Fatores como aumento da carga de trabalho, falta de treinamento, conflito com consumidores, estigma social, exposição involuntária ao risco e ausência de reconhecimento social foram associados ao aumento do estresse ocupacional nesses grupos ^6^ ^,^ ^7^ ^,^ ^28^.

A análise longitudinal demonstrou que, embora os profissionais não relacionados à assistência à saúde tenham iniciado a pandemia com altos riscos em relação à influência no trabalho, houve redução progressiva desse risco, possivelmente associada à maior autonomia percebida no trabalho remoto ^25^. A confiança na gestão apresentou leve melhora em 2021, possivelmente impulsionada pelo maior acesso a recursos e plataformas digitais que se consolidaram ao longo do período. Espera-se que algumas dessas mudanças, como o uso de tecnologias e a flexibilização do trabalho, se mantenham no cenário pós-pandêmico ^29^.

A capacidade para o trabalho manteve-se satisfatória tanto entre profissionais de saúde quanto nos demais, com resultados semelhantes aos períodos anteriores à pandemia. Isso sugere a resiliência dos trabalhadores brasileiros, mesmo diante das adversidades significativas ^30^. Apesar disso, os profissionais de saúde enfrentaram riscos psicossociais persistentes ao longo de todo o período estudado, o que reforça a necessidade de políticas públicas e estratégias institucionais voltadas à proteção e à promoção da saúde mental dos trabalhadores.
